# Complications of Hyaluronic Acid and Calcium Hydroxylapatite Fillers—A Comprehensive Narrative Review of Clinical Presentation and Current Management Strategies

**DOI:** 10.1111/jocd.71104

**Published:** 2026-08-05

**Authors:** Natalia Sukmanskaya, Sebastian Cotofana, Michael Alfertshofer

**Affiliations:** ^1^ Laboratoire Revitacare Saint‐Ouen‐l'Aumône France; ^2^ Department of Dermatology Erasmus University Medical Center Rotterdam the Netherlands; ^3^ Centre for Cutaneous Research, Blizard Institute Queen Mary University of London London UK; ^4^ Department of Plastic and Reconstructive Surgery Guangdong Second Provincial General Hospital Guangzhou Guangdong Province China; ^5^ Department of Plastic Surgery Technical University Munich Munich Germany; ^6^ Department of Oral and Maxillofacial Surgery Charité Universitätsmedizin Berlin Berlin Germany

**Keywords:** calcium hydroxylapatite, complication management, facial anatomy, filler complications, hyaluronic acid

## Abstract

**Background:**

Hyaluronic acid (HA) and calcium hydroxylapatite (CaHA) fillers are among the most widely used injectable materials in aesthetic medicine. Although both exhibit favorable safety profiles, concomitant with increasing use comes a broader spectrum of reported complications, ranging from mild, self‐limited reactions to rare but potentially dramatic events.

**Objective:**

To provide a comprehensive narrative review of the clinical presentation and current evidence‐based management strategies for common and uncommon complications associated with HA and CaHA fillers.

**Methods:**

A narrative review of the dermatologic, plastic surgery, ophthalmologic, and aesthetic medicine literature was conducted, focusing on peer‐reviewed studies, systematic reviews, expert consensus statements, and illustrative case reports addressing complications of HA and CaHA fillers. Complications were categorized by timing, pathophysiology, and clinical severity, and management approaches were synthesized accordingly. Relevant English‐language articles were identified through PubMed/MEDLINE and Scopus up to 2025 using combinations of the different search terms supplemented by hand‐searching of reference lists; evidence was weighted qualitatively, giving precedence to consensus statements and larger studies over individual case reports.

**Results:**

HA and CaHA fillers share overlapping complication profiles, including bruising, swelling, vascular occlusion, infection, nodules, granulomas, and delayed inflammatory reactions. Key differences arise from their inherent product properties: HA complications benefit from enzymatic reversibility with hyaluronidase, which underpins established treatment algorithms for vascular occlusion, Tyndall effect, chronic edema, and nodules. In contrast, CaHA‐related complications rely more heavily on prevention, supportive care, and, in select cases, mechanical removal through surgical intervention, as no enzymatic reversal is available. Early recognition and prompt intervention are critical determinants of outcome, particularly in ischemia‐related complications.

**Conclusion:**

HA and CaHA fillers are effective and generally safe filler materials, yet not without risk. A thorough understanding of complication patterns, material‐specific management strategies, and preventive techniques is essential for clinicians performing filler injections. With appropriate training, education, and safety algorithms to manage adverse events, most complications can be treated effectively, thereby maximizing patient safety and satisfaction.

## 1 Introduction

In recent decades, dermal filler injections have become one of the most popular minimally invasive cosmetic procedures worldwide [1, 2]. Hyaluronic acid (HA) fillers and calcium hydroxylapatite (CaHA) fillers are two of the most widely used materials in soft tissue fillers designed for different facial indications such as soft tissue augmentation, volume restoration, and wrinkle correction. HA is a naturally occurring polysaccharide that is formulated into a gel for injection. It provides immediate volume and is valued for its safety and reversibility as it can be enzymatically degraded by hyaluronidase [3, 4, 5]. CaHA is a synthetic filler composed of micron‐sized particles suspended in a carrier gel. It acts as a biostimulator, thereby catering to a dual effect: providing immediate volume but also stimulating collagen production for delayed and longer‐lasting effects [6]. Common to both filler types is their generally favorable safety profile due to high biocompatibility; however, as their use has increased, likewise have reports of complications [7, 8]. Even skilled injectors occasionally encounter adverse events (AE), ranging from mild, self‐limited side effects to rare but severe outcomes [9].

Generally, complications seen with HA‐ and CaHA‐based dermal fillers can be categorized by timing and underlying pathophysiology. Early complications occur immediately or within days of injection and usually result from the injection procedure itself or product placement. In this category, expected minor reactions caused by the mechanistic trauma incurred by the injection itself, such as bruising, swelling, and pain at the injection site are summarized [10]. Yet, also more serious problems such as acute vascular occlusion—either via inadvertent intravascular injection with obturation from the “inside” or extravascular placement adjacent to an artery with compression from the “outside”—can lead to ischemia with necrosis of the subsequent tissue [11]. On the other hand, delayed complications can develop weeks to months (or in some cases even years) after the initial injection and often have an immunologic or infectious component. Delayed issues can include the formation of nodules or granulomas (i.e., foreign body reactions), chronic edema in the treated area, delayed‐onset inflammatory reactions as well as infections often related to biofilm‐forming bacteria [12, 13, 14]. Another way to classify filler‐related complications is by their severity and indirectly also the “need to treat” them: mild effects such as transient bruising or edema typically resolve without intervention, whereas severe complications like tissue necrosis or vision loss require immediate, resolute and aggressive management.

A thorough understanding of filler‐related complications including their expected incidence, clinical presentation, underlying pathophysiological mechanisms, and recommended management strategies remains a cornerstone for *any* clinician performing these treatments. Prompt recognition and appropriate treatment can significantly reduce the risk of dramatic long‐term sequelae and ensure patient safety. Since aesthetic procedures are defined by their elective nature, ensuring greatest levels of safety remains paramount. Herein, we present a comprehensive narrative review of common complications associated with HA‐ and CaHA‐based soft tissue fillers. Each complication category will be discussed in terms of how frequently it occurs, why it happens, how it manifests clinically, and the evidence‐based strategies to manage it.

## 2 Search Strategy and Evidence Synthesis

This article is conceived as a narrative review. Relevant literature was identified through searches of PubMed/MEDLINE and Scopus for articles published up to 2025, using combinations of the search terms “hyaluronic acid,” “calcium hydroxylapatite,” “CaHA,” “dermal filler,” “soft tissue filler,” “complication,” “vascular occlusion,” “nodule,” “granuloma,” “delayed inflammatory reaction,” “complication” and “complication management” in various combinations. Reference lists of key articles and consensus documents were hand‐searched for additional sources. Preference was given to peer‐reviewed original studies, systematic reviews, and expert consensus statements written in English, with illustrative case reports included where higher‐level evidence was lacking, as is often the case for rare complications. No formal quality scoring or quantitative synthesis was undertaken; instead, the evidence was appraised and weighted qualitatively. Given this pragmatic approach, the review is intended to provide a clinically oriented overview rather than an exhaustive or bias‐controlled appraisal of the literature.

## 3 HA Filler Complications

HA‐based soft tissue fillers are generally considered safe and well‐tolerated. An advantage is their temporary nature and the ability to be dissolved enzymatically with hyaluronidase if necessary, thereby making the management of many complications more approachable [3]. Still, HA injections are not without risk. Complications can occur early (i.e., immediately or within days of treatment) or in a delayed fashion (i.e., weeks to months to years). Below, we detail common complications seen with aesthetic HA filler treatments.

## 4 Bruising and Swelling

Transient bruising and swelling represent the most common minor AE following HA filler injections and are inherent to the injection process itself: Mechanical trauma from the passage of a needle or a cannula through the skin and subcutaneous soft tissues naturally disrupt small blood vessels, resulting in localized bleeding and swelling in the treated facial region. Bruising is particularly frequent in highly vascularized regions such as the perioral and periorbital area [15]. Swelling is the pathophysiological consequence from a combination of tissue injury, local inflammatory response, and the physicochemical properties of HA. As a strongly hydrophilic molecule, HA attracts and binds water, which can further amplify postinjection edema [9]. Consequently, at least mild edema is observed in the vast majority of patients treated with HA fillers. The extent and duration of swelling are heavily influenced by the employed injection technique, extent of tissue manipulation, and ultimately the specific composition of the filler formulation used [16]. Swelling may present as diffuse puffiness of the treated region or as localized fullness, often combined with a certain degree of tissue firmness. In the lips, marked edema is common due to the dense vascular supply and loose connective tissue, whereas even minimal swelling in the periorbital region can be highly visible because of thin overlying skin [17, 18].

Clinically, bruising appears as localized skin discoloration in the injection area ranging from red to purple or blue, often becoming more pronounced within the first postinjection day as hemoglobin degradation products readily spread within the tissue before ultimately being resorbed. Most bruises remain small and confined to the injection area, although larger hematomas may develop if a bigger vessel is injured or if the patient is under anticoagulant medication [15, 19]. This AE is hence generally expected and self‐limited. However, careful clinical assessment still remains essential to differentiate uncomplicated post‐procedural edema from early signs of infection. Benign swelling is typically soft, non‐tender, and improves over time. Another indicator of a benign course is the response to upright positioning since intra‐tissue fluid is being resorbed more readily, whereas concerning presentations may involve progressive pain, erythema or discoloration. Management of bruising and swelling following HA filler treatments predominantly entails conservative measures and begins with appropriate patient counseling and expectation management prior to the injection itself. The patients should be informed that such reactions are common and most of the times self‐limited. Bruising typically resolves over 1–2 weeks as extravasated blood is gradually resorbed. Immediate post‐procedural measures such as firm local compression and the application of cold compresses during the first 24–48 h are widely recommended to reduce extravasation of blood in the soft tissues and promote vasoconstriction, thereby reducing associated edema. Patients are generally advised to avoid physical activity and heat exposure (e.g., sauna) during the early posttreatment period, as these factors may trigger vasodilation and exacerbate bruising and swelling. Adjunctive topical agents including arnica, vitamin K, or bromelain have been reported to be used in clinical practice to accelerate bruise resolution. Although, it must be noted that supporting evidence for these measures remains limited. In very rare cases where a large or tense hematoma organizes, early needle aspiration can be considered while the collection remains liquefied before hematoma formation sets in, particularly if discomfort or tissue compression is present. In the majority of cases, reassurance, documentation, and routine follow‐up are sufficient [15, 19].

Postinjection swelling is similarly addressed with supportive measures, including intermittent cold packs during the first day, avoidance of heat exposure and strenuous physical activity, and elevation of the head to facilitate fluid resorption. Swelling typically peaks within 1–2 days after the procedure and then progressively subsides over several days. In cases of more pronounced edema, particularly in the lips, short‐term use of oral antihistamines or nonsteroidal anti‐inflammatory drugs may provide symptomatic relief when not contraindicated. Still, it should be recognized that firmness or mild swelling can persist for 1–2 weeks as HA continues to hydrate and integrate into the surrounding tissue [20, 21].

A distinct and clinically relevant subtype of post‐filler swelling is persistent malar or periorbital edema, most commonly observed after HA injections in the tear trough or upper cheek region. In susceptible individuals, fluid accumulation in this anatomically constrained area can lead to a chronic puffy appearance that extends beyond the immediate posttreatment period and may persist for months. This phenomenon is thought to result from a combination of impaired lymphatic drainage after vigorous injection device (needle or cannula) placement paired with the hydrophilic nature of HA [22, 23, 24]. When persistent or refractory edema is cosmetically significant, the most effective causal treatment is the removal of the filler using hyaluronidase [25]. In milder cases, conservative strategies such as lymphatic drainage massage, head elevation, and dietary sodium reduction may be attempted first. Preventive strategies are therefore critical and include careful and deep placement of filler on the periosteum rather than superficially along with the use of less volume or less hydrophilic HA formulations [26, 27].

## 5 Vascular Occlusion and Ischemic Complications

Vascular occlusion represents one of the most serious and feared complications of filler injections in general and occurs when material is inadvertently introduced into an artery or when extravascular filler compresses an artery sufficiently to impair blood flow to the supplied tissues [11]. Although vascular complications are rare, with reported incidences below 0.1%, the risk is markedly higher in anatomically risky regions such as the glabella, forehead and nose, where direct anastomoses with the ophthalmic artery as a branch of the internal carotid artery can be observed. In these locations, occlusion may result in skin necrosis if cutaneous arterial branches are affected and, in severe cases, permanent vision loss if the central retinal artery is being occluded [7, 8]. Risk factors include greater volumes in bolus injections, high injection pressure, limited anatomical awareness, and the use of sharp needles in highly vascular regions. Nevertheless, inadvertent arterial penetration may still occur despite standard precautionary measures, meticulous technique and good anatomical knowledge due to the highly variable nature of facial vessels [28, 29].

Pathophysiologically, the intravascular or extravascular HA filler hinders sufficient perfusion of subsequent tissues, either via obturation or compression, respectively. However, another potential mechanism discussed is the limitation of blood flow through arterial vasospasm—with vasoconstriction—triggered by the mechanical trauma of the injection [30]. In all scenarios, compromised perfusion rapidly deprives tissues of essential oxygen and nutrients, resulting in acute ischemic injury that may progress to necrosis of affected tissues if arterial supply is not promptly and sufficiently restored. Clinically, vascular occlusion typically presents during or immediately after injection with sudden, disproportionate pain and localized blanching, often resulting in a reticular “net‐like” pattern corresponding to the affected vascular territory. Without reperfusion, the skin is ultimately set to display blistering, eschar formation, and ulceration. While transient blanching due to vasospasm or local anesthetic effects may occur, true vascular occlusion is characterized by persistent pain and color change and must be treated as an emergency [31, 32]. Involvement of vessels communicating with the ophthalmic circulation may manifest as visual disturbances or sudden vision loss, constituting an ophthalmologic emergency.

Management of HA‐related vascular occlusion centers around immediate enzymatic degradation with hyaluronidase using a high‐dose, pulsed approach guided by close clinical follow‐up. In the CMAC guideline, hyaluronidase is reconstituted at high concentration (1500 units in 1 mL of bacteriostatic 0.9% sodium chloride or 1%–2% lidocaine) and infiltrated by needle or cannula along the course of the affected artery as well as across the entire ischemic area. An often reported clinical indicator for perfusion of the affected area is the capillary refill time: if it remains delayed with a cut‐off of typically > 3 s, the protocol is repeated, with redosing performed frequently until clinical improvement is observed. The guideline further notes that intra‐arterial injection is not required—often also not verifiable in the humble opinion of the authors—since hyaluronidase is thought to diffuse into the vessel lumen in extravascular administration [33]. The rationale for repeated high‐dose administration is to ensure continuous enzymatic exposure of intravascular and perivascular HA, thereby reducing the risk of partial degradation and distal embolization of fragmented filler. Previously, DeLorenzi has noted this clinical need by proposing a high‐dose pulsed hyaluronidase approach in which relatively large, tissue‐volume‐based doses are reinjected at approximately hourly intervals until clinical resolution of ischemia is achieved, assessed by normalization of skin color and capillary refill time and the absence of pain [34]. Adjunctive measures commonly reported in the literature alongside causal hyaluronidase injections include the local application of warm compresses to promote vasodilation and gentle massage to facilitate dispersion of filler material, while simultaneously avoiding excessive pressure that could propagate further embolization. Antiplatelet therapy with acetylsalicylic acid (e.g., 300–325 mg orally as a stat dose with 75–100 mg daily until reperfusion) is frequently described as an adjunct treatment aiming to reduce secondary thrombus formation and support microcirculatory flow [33, 35]. Topical 2% nitroglycerin paste on the affected skin area or sublingual nitroglycerin tablets (0.6 mg) have been described as a local vasodilatory adjunct, although systemic absorption and hypotension must be considered, therefore being upon the physician's discretion in light of other diagnoses [35, 36]. Short courses of systemic corticosteroids, such as oral prednisone in the range of 20–40 mg daily for 3–5 days, have been described to mitigate inflammation and edema that may further compromise perfusion [31, 35]. Hyperbaric oxygen therapy (HBOT) is another option with reported protocols typically involving daily sessions at 2.5 atm absolute for 90 min, particularly when initiated within the first 24–48 h of ischemic injury [37, 38]. Where available, point‐of‐care high‐frequency and doppler‐ultrasound has become an increasingly valuable adjunct in this setting, allowing the occluded vessel and residual filler to be visualized directly, guiding targeted infiltration of hyaluronidase, and permitting real‐time confirmation of restored flow; its use may reduce the total enzyme dose required and remove some of the guesswork from serial reassessment [39, 40].

When treatment is started early and aggressively, restoration of perfusion, as evidenced by return of normal skin coloration and pain reduction, may effectively prevent progression to necrosis. Hyaluronidase administration ought to be repeated in the following days if a persistence of clinical ischemia signs is noted. Once tissue necrosis has developed, management shifts toward wound care, including topical antimicrobial therapy, atraumatic dressings, and monitoring for secondary infection, recognizing that healing may require weeks and often results in permanent scarring [41].

In cases of vision loss following HA filler injection, emergency ophthalmologic management is required. Reported interventions include immediate ocular massage, intraocular pressure reduction with agents such as acetazolamide, anterior chamber paracentesis, and systemic corticosteroids. Retrobulbar hyaluronidase injections, using doses ranging from several hundred to over 1500–3000 units, have been described in isolated case reports. However, for the aforementioned measures, strong compelling evidence for visual recovery remains limited. HBOT has also been reported in this context, though successful restoration of vision is rare. Consequently, prevention through cautious technique, avoidance of high‐risk injection patterns, and immediate access to hyaluronidase remains paramount [42, 43, 44].

Although most vascular occlusions declare themselves immediately, delayed ischemic presentations occurring several hours to days after injection have been described, potentially due to progressive vascular compromise or secondary thrombosis caused by the filler material [45, 46]. Patients should be instructed to report any delayed pain or skin changes immediately, as delayed presentations warrant the same urgent evaluation and intervention as events observed directly after the causative injection.

## 6 Tyndall Effect and Skin Discoloration

The Tyndall effect refers to a bluish discoloration of the skin that is more likely to occur when HA filler is placed too superficially. It is caused by a change of light scattering through the translucent HA gel, whereby shorter blue wavelengths are reflected back to the observer, producing the characteristic discoloration. This phenomenon is most commonly observed in areas with thin and delicate skin, which is prominently found in the tear trough [47]. Although its true incidence is not well quantified, it is generally regarded as an infrequent complication and is primarily related to inappropriate injection depth (i.e., too superficial) and inadequate product choice (i.e., high molecular weight). Even small volumes of superficially deposited HA can result in visible discoloration in predisposed anatomical regions. Clinically, this effect presents as a persistent, localized blue or slate‐gray hue beneath the skin that does not evolve or fade like a bruise and remains stable as long as the HA is present in the treated area. Often patients report a worsening of periorbital dark circles following tear trough treatment rather than the initially desired aesthetic improvement [48].

Management is straightforward, as the Tyndall effect is a purely aesthetic issue rather than a pathologic complication. The treatment of choice is the removal of the causative material via enzymatic degradation using hyaluronidase. In most cases, relatively small doses are sufficient, commonly in the range of 30–75 units injected directly into the affected area with a fine needle, depending on the volume of the superficial deposit. Visible improvement typically occurs within 24–72 h as the HA gel is degraded and cleared [49, 50]. In selected cases, partial dissolution may be opted for to reduce discoloration while preserving some volumizing effect in the tear trough region. Observation alone or cosmetic cover‐up is an alternative option for very mild cases, as HA fillers gradually degrade over months and the discoloration fades.

Prevention remains central and relies on appropriate product selection and injection technique. Deep placement of filler, particularly in the supra‐periosteal plane in the tear trough, use of lower‐viscosity and less light‐reflective HA formulations and conservative volumes reduce the risk. Other filler‐related discolorations, such as transient bruising or whitish sheen from extremely superficial deposits, should be distinguished from the true Tyndall effect [50, 51]. Of note, this phenomenon is specific to translucent fillers such as HA and is not observed with opaque materials such as CaHA.

## 7 Infection and Inflammatory Reactions

Infectious complications following HA filler injections tend to be uncommon, reflecting the use of aseptic technique and the good biocompatibility of HA. Still, when they occur, they may present either acutely or in a delayed fashion. Acute infections typically arise within the first week after injection and result from inoculation of microorganisms at the time of treatment, leading to localized cellulitis or abscess formation [52]. On the other hand, delayed presentations have historically been attributed, at least in part, to bacterial biofilms forming on the filler material; however, direct clinical evidence for biofilm remains scarce, as cultures are frequently negative and histopathologic confirmation is rarely reported [53, 54]. Such delayed presentations are therefore better regarded as part of a broader spectrum of delayed (or late) inflammatory reactions (DIRs/LIRs), in which different factors come into play: the physicochemical properties of the filler, the host immune status, and low‐grade bacterial contamination may act synergistically as triggers, rather than as a distinct, purely biofilm‐mediated entity. Common organisms implicated include skin commensals such as *Staphylococcus aureus* or *epidermidis*, which may remain dormant for weeks or months before triggering an inflammatory flare‐up [55].

Clinically, acute infection presents with progressive erythema, local warmth, swelling, and tenderness at the injection site, typically worsening rather than improving over time if not targeted. Fluctuance or purulent drainage suggests abscess formation, and systemic symptoms such as general malaise or low‐grade fever may occur in more extensive cases. In contrast, delayed biofilm‐related infections usually present weeks to months after treatment as firm nodules within previously injected areas, often accompanied by recurring localized erythema and tenderness. Simultaneous inflammation of several previously treated sites, particularly following a systemic immune stimulus such as infection or vaccination, is characteristic of a delayed inflammatory reaction but does not by itself establish an underlying biofilm [52, 55].

Management vastly depends on timing and severity. Acute postinjection infections should be treated promptly with empiric oral antibiotics targeting common skin flora, such as amoxicillin‐clavulanate, a first‐generation cephalosporin, or clindamycin in penicillin‐allergic patients. According to the latin proverb “ubi pus, ibi evacua,” if an abscess is present, needle aspiration or incision and drainage is always required, as antibiotics alone are insufficient. Generally, infections should be approached “broad” first (i.e., empiric antibiotic regimen), and after identifying the causative bug through bacterial culture, with a more “targeted” regimen. Adjunctive hyaluronidase may be used to dissolve the nidus of the infection and facilitate immune clearance, particularly when infection is persistent or localized to a filler pocket [56, 57].

As an *ultima ratio*, refractory or recurrent lesions may ultimately be accessible only through surgical excision, particularly when well‐formed granulomas or chronically infected filler deposits persist [35]. Culture and sensitivity testing of aspirated material or excised tissue is strongly recommended to guide targeted antimicrobial treatment, although cultures may not uncommonly be negative in delayed inflammatory reactions. Close follow‐up is essential to monitor clinical response and adjust therapy as needed [58]. Following resolution, reinjection of the affected area should be approached cautiously, with many clinicians avoiding retreatment or selecting alternative products and techniques.

## 8 Granulomas and Delayed Nodules

True granulomatous reactions following HA filler injections are rare but represent an important category among delayed complications. Together with the delayed presentations discussed above, granulomas can be understood not as separate diagnoses but as points along a single spectrum of DIRs/LIRs: the same reaction may be labeled a delayed nodule, a granuloma, or a biofilm‐associated flare depending on which feature predominates and on whether histology is obtained, and in routine practice the specific etiology often cannot be determined with absolute confidence at presentation [12, 59, 60]. Granulomas are defined as a chronic inflammatory responses characterized by collections of macrophages, multinucleated giant cells, and other immune cells surrounding a foreign material [16]. With modern HA fillers, reported incidences of histologically confirmed foreign body granulomas are very low, generally estimated between approximately 0.02%–0.4% for delayed nodules overall, recognizing that not all such nodules represent the histologic entity of true granulomas [61]. Nevertheless, given the large global volume of HA filler injections, even rare events translate into a clinically relevant number of affected patients. Granulomas typically manifest months to years after injection, and their pathogenesis is likely multifactorial, involving individual immune predisposition, product composition, and immune activation triggered by systemic or local inflammatory events (e.g., dental procedures) [57, 61, 62].

It is essential to distinguish true granulomas from noninflammatory filler nodules. Not all delayed lumps represent immune‐mediated reactions as some reflect localized filler accumulation, fibrosis, or scar formation related to injection technique [16, 63]. These non‐granulomatous nodules often appear earlier, within weeks to months, are usually non‐tender and non‐erythematous, and primarily constitute an aesthetic concern. In clinical practice, however, differentiation between mechanical nodules and early granulomatous reactions may be difficult without histologic confirmation, and the term “nodule” is often used pragmatically until response to treatment clarifies the underlying pathophysiological process [64]. Clinically, patients often note a change from a previously soft and smooth aesthetic result to localized firmness and “bumpiness.” Without intervention, granulomas rarely resolve spontaneously and may slowly enlarge or multiply over time as inflammation persists.

Management focuses on removal of the causative material, suppression of the immune response, and treatment of any associated infection. Hyaluronidase is generally the first‐line intervention, injected directly into the affected nodules to dissolve residual HA and reduce the antigenic stimulus. Multiple treatment sessions may be required, particularly in longstanding lesions where filler is partially encapsulated by fibrotic tissue. Intralesional corticosteroids, most commonly triamcinolone acetonide, are frequently used to attenuate the immune response (i.e., macrophage activity) and soften fibrotic capsules. To minimize risks of skin atrophy or dyspigmentation, repeated low‐dose injections spaced weeks apart are preferred over one‐time high‐dose treatments [3, 64, 65]. Additional adjuncts reported in refractory granulomas include intralesional 5‐fluorouracil (5‐FU), particularly when fibrotic components predominate, although it needs to be noted that this approach is more commonly seen in reactions to fillers aiming for biostimulatory processes [66]. When infectious contribution cannot be excluded, such as in the presence of fluctuation or abscess formation, antibiotic therapy should be incorporated alongside hyaluronidase and corticosteroids. Given the difficulty in reliably distinguishing sterile granulomas from biofilm‐associated inflammation, combined antibiotic and anti‐inflammatory strategies are frequently adopted [64, 65, 66, 67]. As an *ultima ratio*, surgical excision is reserved for granulomas that remain persistent or progressively symptomatic despite prolonged medical management. Excision or incision and curettage can provide definitive resolution but carries a risk of scarring and is typically limited to localized lesions in anatomically acceptable areas. Whenever tissue is removed or aspirated, histopathologic and microbiologic evaluation is recommended to confirm diagnosis and guide further management [68]. Where available, imaging can further aid this assessment: on high‐frequency ultrasound, filler deposits appear as well‐defined “anechoic pockets,” whereas granulomas and fibrosis produce a more heterogeneous, ill‐defined signal, and in the same session direct intralesional injection can be performed [69, 70]. Patients ought to be counseled that granuloma treatment is often protracted and may require multiple interventions over several months. A prior granulomatous reaction does not necessarily preclude future HA filler use, though cautious retreatment with alternative products or test dosing is advisable [71].

The herein discussed complications and their respective management approaches are summarized in Table 1.

**TABLE 1:** Summary of complications associated with HA‐based soft tissue filler treatments and their management strategies.

Complication	Key presentation	First‐line management	Alternative/adjunct treatment/prevention
Bruising/hematoma	Local discoloration within 24 h	Compression + cold packs (24–48 h), reassure patient, avoid heat/strenuous activity	Rare: aspiration if large/tense hematoma formation
Injection‐related swelling	Soft puffiness, peaks 1–2 days	Cold packs, head elevation, avoid heat/strenuous activity	Antihistamine/NSAID short‐term if needed; differentiate from infection (progressive pain/erythema)
Persistent malar/periorbital edema	Prolonged puffiness after tear trough/upper cheek	Hyaluronidase if persistent/cosmetically significant	Conservative trial (lymph drainage, head elevation, ↓salt) if mild; prevent with deep placement + low volumes/less hydrophilic HA
Vascular occlusion (ischemia/necrosis risk)	Sudden severe pain, blanching/livedo, delayed capillary refill	High‐dose pulsed hyaluronidase, infiltrate entire ischemic area; reassess + repeat (often hourly) until reperfusion	Warm compress + gentle massage; aspirin often used; nitroglycerin (caution), short steroids; consider HBOT early; necrosis → wound care
Ocular embolization/vision loss	Visual disturbance/monocular vision loss	Immediate emergency ophthalmology	Ocular massage, IOP lowering, paracentesis; retrobulbar hyaluronidase/HBOT reported but limited benefit; prevention is key
Tyndall effect	Persistent blue‐gray hue (superficial HA, especially tear trough)	Hyaluronidase (usually small doses)	Partial dissolution optional; prevent with correct depth + conservative volumes
Acute infection (days)	Worsening erythema, warmth, tenderness; abscess fluctuant	Empiric oral antibiotics (skin flora)	Abscess → aspiration/I&D + culture → targeted therapy; consider hyaluronidase if persistent nidus
Delayed infection/biofilm	Weeks–months; tender erythematous nodules; multiple sites may flare	Antibiotics + hyaluronidase; close follow‐up	Hyaluronidase to remove nidus; refractory → excision; cultures may be negative
Delayed nodules/granulomas	Months–years; firm nodules ± erythema/tenderness	Hyaluronidase (often multiple sessions) + intralesional corticosteroids	Add antibiotics if biofilm cannot be excluded; refractory → 5‐FU (selected cases) or surgical excision

## 9 Calcium Hydroxylapatite (CaHA) Filler Complications

CaHA fillers are considered a semipermanent or long‐lasting filler, with effects lasting around 12–18 months as they show the added effect of stimulating collagen production in the injected area. CaHA filler injections can also lead to complications, similar to those seen with HA and some unique to its composition. One major difference is that CaHA is not dissolvable with any readily available enzyme such as hyaluronidase for HA fillers. This means that certain complication management strategies differ for CaHA.

## 10 Bruising and Swelling

As with any injectable filler, CaHA injections are commonly associated with transient bruising and swelling due to the mechanical trauma inflicted by the injection itself. The frequency of these minor adverse effects is comparable to other dermal fillers [72, 73]. Because CaHA is typically placed in deeper tissue planes and often delivered using larger‐gauge needles or cannulas, particularly in areas such as the cheeks or jawline, there is a theoretical increased risk of vascular trauma. In practice, however, careful technique, use of cannulas, slow injection, and meticulous anatomical knowledge largely mitigate this risk [74]. Immediately after treatment, localized swelling and firmness are common and reflect both the injected volume and the presence of the carrier gel. This firmness typically resolves over a few days as the carrier integrates alongside tissue adaptation.

Management of bruising and swelling following CaHA injection is conservative and parallels that previously described for HA fillers: Early application of cold compresses, head elevation, and avoidance of blood‐thinning medications—when clinically feasible—are among the standard armamentarium of measures. Adjuncts such as *Arnica montana* or bromelain are frequently recommended, although supporting evidence is limited. Patients should be reassured that a sensation of fullness or stiffness during the first week is expected and is typically expected to limit as the product settles [15, 19]. Routine massage is generally unnecessary and not encouraged unless a local irregularity is prominent shortly after injection. While CaHA cannot be enzymatically dissolved, early contour irregularities may sometimes be improved with careful manual redistribution. In the rare event of a significant hematoma, management consists of observation and, in rare cases, aspiration of a formed hematoma once the collection is accessible. Bruising typically resolves within 1–2 weeks and can be effectively camouflaged with makeup.

## 11 Vascular Occlusion and Ischemia

Although much attention has focused on HA fillers, injections employing CaHA‐based fillers likewise carry a risk of intravascular injection and ischemic complications [75]. Any viscous filler introduced into an arterial circulation can cause vascular occlusion, and CaHA poses no exception [76]. While severe events such as skin necrosis or vision loss are rare, documented cases exist, with a systematic review by Fortes et al. reporting serious complication rates at 0.27% comprising, but not limited to, cases of skin necrosis [72]. Historically, perceived risk may have been lower because CaHA was used more conservatively in high‐risk regions such as the glabella and nasal dorsum. Still, cases of skin necrosis and ocular embolization following CaHA injection have been reported [77, 78]. Once intravascular, CaHA behaves similarly to HA in its clinical consequences, producing acute interruption or limitation of blood flow with subsequent tissue ischemia and hypoxia. Its particulate composition, however, may contribute to more persistent mechanical obstruction and local inflammatory response. The clinical presentation of CaHA‐related vascular occlusion mirrors that seen with HA fillers: Patients typically experience sudden, strong pain and visible blanching of the skin within the vascular territory supplied by the affected artery, followed by mottled discoloration if perfusion is not restored immediately [75, 77]. Depending on the vessel involved, blanching may extend beyond the injection site, and in rare cases involving the ophthalmic circulation, visual disturbance or acute vision loss may occur. While delayed recognition is possible in partial or distal occlusions, acute pain and color change during injection remain key warning signs.

Management of CaHA‐induced vascular occlusion is inherently more challenging than that of HA since no enzymatic agent can dissolve CaHA microspheres. Immediately stopping the injection and thorough clinical assessment are essential. Supportive measures are directed at limiting ischemic injury and promoting collateral perfusion and can comprise gentle massage and application of warmth to encourage vasodilation [76]. Although hyaluronidase does not degrade CaHA, some consensus guidelines still describe its use in the affected area, either to address any concomitant HA component in the area or to improve tissue permeability and reduce edema and inflammation [76, 79, 80]. Of note, Danysz et al. as well as Robinson noted in their preclinical experimental studies that the use of calcium‐chelating agents such as sodium thiosulfate resulted in a volume reduction of CaHA, but evidence is extremely limited and such interventions remain off‐label and rescue‐based [81, 82]. Antiplatelet therapy with aspirin is commonly employed to potentially reduce thrombus formation, and additional agents such as low‐molecular‐weight heparin or pentoxifylline may additionally be tried to support microcirculatory flow. HBOT represents an important adjunct, particularly when initiated early, as it may improve tissue oxygenation and reduce the extent of necrosis in the absence of a means to remove the embolus [76, 83].

If ischemia progresses to necrosis, analogous to HA filler‐induced necrosis, the management transitions to wound care, including protection of devitalized tissue, infection prevention, surgical debridement when indicated, and long‐term scar management. Surgical removal of CaHA material has been attempted in isolated cases but is rarely feasible because of rapid dispersion of microspheres within the tissue [76, 83]. In ocular embolization caused by CaHA, therapeutic options are theoretically even more limited than with HA, as the material cannot be enzymatically dissolved. Management therefore relies on emergency ophthalmologic measures such as ocular massage, reduction of intraocular pressure, systemic anti‐inflammatory therapy, and vasodilatory support, with HBOT used as an adjunct in selected cases [84, 85]. Notably, Liu and colleagues reported a rare case of near‐complete visual recovery following CaHA‐induced vision loss after an injection in the nasal dorsum, achieved through early administration of intravenous corticosteroids and prostaglandin E1, followed by adjunctive HBOT, highlighting that partial recovery may be possible when embolization predominantly affects the anterior ocular circulation rather than the central retinal artery [78]. Nevertheless, overall visual prognosis remains poor, and full recovery has not been reliably reproduced across many cases. Consequently, prevention is paramount, and CaHA should be used with particular caution in high‐risk regions including the forehead, glabella, and nose, employing slow, low‐pressure injections, small aliquots, and techniques that minimize intravascular entry [7, 8].

## 12 Nodules and Superficial Deposits

Nodule formation represents one of the most frequently reported complications associated with CaHA fillers. Among patients who develop palpable nodules, the majority are self‐limiting and asymptomatic. Such nodules are primarily related to the particulate product nature of CaHA and to an injection technique targeted at a more superficial plane. When CaHA is placed in highly mobile regions or insufficiently distributed within the tissue, microspheres may cluster or induce focal collagen overproduction, resulting in localized firmness [86]. For this complication, the perioral region has been noted to be particularly susceptible with unacceptably high rates of firm nodules [87]. Superficial placement of CaHA may further result in visible whitish or chalky deposits due to the product's opaque composition, underscoring the importance of deep subdermal or supraperiosteal placement [88]. Clinically, CaHA nodules may present as non‐tender subdermal lumps that are only noticeable on palpation and often soften over time as the carrier gel is resorbed and collagen remodeling occurs [88]. Less commonly, nodules may become inflamed, presenting weeks to months after injection with firmness, erythema, and tenderness, reflecting a granulomatous or low‐grade inflammatory response. True foreign‐body granulomas to CaHA are rare but have been reported and behave similarly to those seen with HA fillers, albeit without the option of enzymatic dissolution [76, 83, 89]. Analogous to the HA‐induced counterpart, granulomas after CaHA‐based filler injections required histologic confirmation for their diagnosis.

Management heavily depends on onset, symptom severity, and cosmetic impact. Asymptomatic, non‐visible nodules are often best managed expectantly, as many resolve gradually over time. Early, gentle massage may help redistribute product if detected shortly after injection, but established nodules rarely respond to manual manipulation. For visible or cosmetically disturbing nodules, intervention may be required. Because CaHA cannot be enzymatically degraded, approaches focus on mechanical removal or dispersion, including needle puncture, saline injection, gentle massage, or mechanical vibration. Since CaHA is strongly hyperechoic by nature and therefore produces posterior acoustic shadowing, high‐frequency ultrasound is particularly helpful for confirming the diagnosis and mapping the extent and depth of a deposit [70]. This characteristic is especially advantageous for CaHA, since the material—unlike HA—cannot be dissolved enzymatically: precise sonographic localization allows mechanical interventions such as needle puncture, saline dispersion, or targeted excision to be directed in a more targeted fashion while sparing surrounding tissue, ultimately improving their effectiveness and reducing collateral trauma. Sodium thiosulfate has been described in isolated reports as a chemical approach to dissolve calcium deposits, typically injected intralesionally in small volumes and repeated over several sessions, although supporting evidence remains anecdotal [86, 90].

Therefore, prevention relies on appropriate patient selection, deep injection planes, avoidance of high‐risk areas such as the perioral and periorbital region, the use of conservative volumes, and skillful application of the product. Early recognition of small irregularities allows timely intervention and may prevent progression to fixed nodules.

## 13 Granuloma Formation (Delayed Reactions)

Although CaHA is generally considered bioinert, infectious and granulomatous reactions can still occur, albeit infrequently. Published data suggest that delayed reactions such as granulomas occur in fewer than 1% of treated patients [72]. When granulomas do develop, their clinical behavior closely resembles that observed with HA fillers, typically presenting as firm subcutaneous masses months after injection. Because CaHA is long‐lasting and cannot be enzymatically degraded, granulomatous reactions may arise even years later if immune activation occurs, effectively extending the window during which host‐material interactions can trigger chronic inflammation.

Clinically, CaHA‐associated granulomas present as firm, sometimes multiple nodules at prior injection sites and may be accompanied by erythema, tenderness, or swelling during active inflammatory phases. These lesions often demonstrate a fluctuating course, with periods of relative quiescence punctuated by inflammatory flares, sometimes following systemic immune stimuli such as intercurrent infection [62]. Compared with simple mechanical nodules, granulomas are typically larger, less well circumscribed, and more inflammatory, and in some cases progress to significant fibrosis [76, 83]. Granulomas occurring in anatomically sensitive regions, particularly the periorbital area, may extend into deeper tissue planes and cause functional impairment, underscoring the importance of early recognition and structured management.

Treatment of CaHA granulomas is inherently more complex than that of HA‐related granulomas and centers on immunomodulation rather than material dissolution. Repeated intralesional corticosteroid injections are the mainstay of therapy. Triamcinolone acetonide is most commonly used, typically at low to moderate concentrations tailored to lesion size and anatomical location, and administered at intervals of approximately 4–6 weeks [76, 86, 91]. The goal is gradual suppression of macrophage‐driven inflammation and remodeling of fibrotic tissue rather than rapid resolution, as overly aggressive dosing increases the risk of cutaneous atrophy, telangiectasia, or hypopigmentation, particularly in thin skin regions [92]. Multiple treatment sessions are often required, and clinical improvement may be incremental.

When granulomas are extensive, symptomatic, or inadequately responsive to intralesional therapy, systemic corticosteroids may be introduced, usually as a short oral taper designed to dampen active inflammation while minimizing long‐term adverse effects. In parallel, many clinicians elect to prescribe prolonged courses of antibiotics, typically for 2–4 weeks, when a biofilm‐associated inflammatory component cannot be confidently excluded, even in the absence of overt infection [52, 66]. In rare, treatment‐resistant cases, additional immunomodulatory agents have been reported, including janus kinase inhibitors (i.e., abrocitinib) or low‐dose systemic immunosuppressants such as methotrexate, extrapolated from management strategies for sarcoidosis or other granulomatous disorders [93, 94]. These interventions remain anecdotal and are reserved for exceptional circumstances under specialist supervision. Surgical excision represents the definitive option for persistent, symptomatic CaHA granulomas that fail medical management. Excision may be facilitated by the radiopaque nature of CaHA on imaging, allowing more precise localization of deposits; however, surgery carries an inherent risk of scarring and is generally reserved for well‐demarcated lesions in surgically favorable locations [76, 83, 86].

## 14 Chronic Edema

CaHA injected in anatomically delicate regions may occasionally result in prolonged edema and tissue induration. This has been most clearly illustrated in reported cases involving off‐label CaHA placement in the lower eyelid or tear trough, where patients developed persistent periorbital swelling and firmness that failed to resolve with time [88, 89]. The underlying mechanism is likely multifactorial, involving mechanical impairment of lymphatic drainage by the injection procedure itself, secondary fibrosis within the soft tissues, and a low‐grade chronic inflammatory response that increases vascular permeability and promotes fluid retention.

Management of CaHA‐associated chronic edema depends on severity and duration. Initial measures may include short courses of systemic or intralesional corticosteroids to address any inflammatory component and close observation to allow for tissue remodeling as the carrier gel resorbs [35, 79, 88]. When edema and induration persist despite these measures, particularly in functionally sensitive areas such as the lower eyelid, surgical removal of the filler and associated fibrosis becomes the definitive treatment. Although such complications are uncommon, reflecting general avoidance of CaHA in edema‐prone regions, early recognition and intervention are important, as chronic edema and fibrosis in the periorbital area may lead to lasting aesthetic distortion or functional impairment. Surgical management also allows histopathologic confirmation, which typically demonstrates granulomatous inflammation and fibrosis surrounding CaHA microspheres, thereby clarifying the underlying pathophysiology.

The herein discussed complications and their respective management approaches are summarized in Table 2.

**TABLE 2:** Summary of complications associated with CaHA‐based soft tissue filler treatments and their management strategies.

Complication	Key presentation	First‐line management	Escalation/notes
Bruising/swelling	Postinjection edema, firmness (often deeper planes), possible hematoma	Cold packs, head elevation, reassurance	Rare: aspiration of large hematoma; early gentle redistribution if immediately recognized irregularity
Vascular occlusion (ischemia/necrosis)	Sudden severe pain, blanching, livedoid pattern; possible skin necrosis; rare ocular events	Immediate stop injection, warm compress, gentle massage, urgent assessment	No enzymatic reversal; aspirin commonly used; consider LMWH/pentoxifylline (limited evidence); HBOT early; hyaluronidase sometimes used adjunctively (not dissolving CaHA); necrosis → wound care
Ocular embolization	Visual disturbance/acute vision loss	Immediate ophthalmology emergency management	Ocular massage, IOP reduction, steroids; HBOT adjunct; prognosis generally poor; prevention critical
Noninflammatory nodules	Palpable subdermal lumps (often asymptomatic); superficial placement → whitish deposits	Observation (many soften over time)	Early massage if very recent; saline dilution + massage; needle puncture/expression; mechanical vibration reported
Superficial visible deposits	Chalky/white appearance under thin skin	Mechanical reduction techniques	Intralesional saline dilution; sodium thiosulfate described (limited evidence)
Inflammatory nodules/granulomas	Months–years; firm, erythematous, tender nodules; may fluctuate	Intralesional corticosteroids (repeated, low‐moderate dose)	Add antibiotics if biofilm cannot be excluded; short systemic steroids for flares; rare: methotrexate/JAK inhibitors (case‐based); surgical excision if refractory
Chronic edema (esp. periorbital, off‐label)	Persistent swelling, induration	Short course steroids (systemic or intralesional)	Persistent cases → surgical removal; prevention: avoid edema‐prone regions

## 15 Conclusion

Both, HA and CaHA fillers are widely used and generally safe tools for facial rejuvenation and beautification, however, both are associated with a spectrum of early and delayed complications. While many adverse events are mild and self‐limited, serious outcomes such as vascular occlusion, infection, or granulomatous reactions require prompt recognition and structured management. The reversibility of HA with hyaluronidase provides a key safety advantage and underpins many evidence‐based treatment algorithms, whereas complications related to CaHA rely more heavily on prevention, early detection, and supportive care due to the absence of a direct reversal agent. For delayed complications, particularly nodules and inflammatory reactions, a pragmatic approach that considers infectious, immune‐mediated, and mechanical causes is essential. Patient education, informed consent, and routine follow‐up further contribute to early detection and favorable outcomes. With thorough anatomical knowledge, careful technique, and preparedness to manage complications, clinicians can minimize risk and safely integrate dermal fillers into aesthetic practice as evidence‐based guidance continues to evolve.

## Funding

The authors have nothing to report.

## Conflicts of Interest

The authors declare no conflicts of interest.

## Data Availability

The authors have nothing to report.
